# Efficacy of Intra-Operative Topical Wound Anaesthesia to Mitigate Piglet Castration Pain—A Large, Multi-Centred Field Trial

**DOI:** 10.3390/ani11102763

**Published:** 2021-09-22

**Authors:** Meredith Sheil, Giulia Maria De Benedictis, Annalisa Scollo, Suzanne Metcalfe, Giles Innocent, Adam Polkinghorne, Flaviana Gottardo

**Affiliations:** 1 Animal Ethics Pty. Ltd., Yarra Glen, VIC 3775, Australia; 2 Department of Animal Medicine, Production and Health, Padova University, Viale dell’Università 16, 35020 Legnaro, Italy; giuliamaria.debenedictis@unipd.it (G.M.D.B.); flaviana.gottardo@unipd.it (F.G.); 3 Department of Veterinary Sciences, University of Torino, Largo Braccini 2, 10095 Grugliasco, Italy; annalisa.scollo@unito.it; 4 Knoell Animal Health Ltd., Bank Barn, How Mill, Brampton CA8 9JY, UK; smetcalfe@knoell.com; 5 Biomathematics and Statistics Scotland, Edinburgh EH9 3FD, Scotland, UK; giles.innocent@bioss.ac.uk; 6 Department of Microbiology and Infectious Diseases, NSW Health Pathology, Nepean Hospital, Penrith, NSW 2750, Australia; adam@majormitchellconsulting.com.au or; 7 Major Mitchell Consulting, Buderim, QLD 4556, Australia

**Keywords:** lidocaine, bupivacaine, adrenaline, peri-operative, topical, anaesthetic, related behaviour, vocalisation

## Abstract

**Simple Summary:**

Piglet castration causes pain and stress to the animal. Although desperately needed, there are complexities developing safe and effective methods of pain alleviation applicable for on-farm use. Topical anaesthesia, instilled to the wound during surgery, is a newly evolving on-farm method to mitigate castration pain. In the current study, we investigated the use of Tri-Solfen^®^ (Animal Ethics Pty Ltd, Melbourne, Australia), a topical local anaesthetic and antiseptic formulation, instilled to the wound during the procedure, to alleviate subsequent castration-related pain experienced in piglets. We performed a large, blind, multi-centred trial comparing pain in piglets castrated with or without Tri-Solfen^®^. Piglets treated with wound instillation of Tri-Solfen^®^, 30 s prior to subsequent castration, showed significantly lower pain-induced motor and vocal responses during the procedure. Acute post-operative pain-related behaviours, evident in piglets in the first 30 min following castration, were also significantly reduced in treated piglets compared with untreated piglets. Using this method, Tri-Solfen^®^ provides an effective on-farm method to mitigate acute castration-related pain in young piglets.

**Abstract:**

Piglet castration results in acute pain and stress to the animal. There is a critical need for effective on-farm methods of pain mitigation. Local anaesthesia using Tri-Solfen^®^ (Animal Ethics Pty Ltd., Melbourne, Australia), a topical local anaesthetic and antiseptic formulation instilled to the wound during surgery, is a newly evolving on-farm method to mitigate castration pain. To investigate the efficacy of Tri-Solfen^®^, instilled to the wound during the procedure, to alleviate subsequent castration-related pain in neonatal piglets, we performed a large, negatively controlled, randomised field trial in two commercial pig farms in Europe. Piglets (173) were enrolled and randomised to undergo castration with or without Tri-Solfen^®^, instilled to the wound immediately following skin incision. A 30 s wait period was then observed prior to completing castration. Efficacy was investigated by measuring pain-induced motor and vocal responses during the subsequent procedure and post-operative pain-related behaviour in treated versus untreated piglets. There was a significant reduction in nociceptive motor and vocal response during castration and in the post-operative pain-related behaviour response in Tri-Solfen^®^-treated compared to untreated piglets, in the first 30 min following castration. Although not addressing pain of skin incision, Tri-Solfen^®^ is effective to mitigate subsequent acute castration-related pain in piglets under commercial production conditions.

## 1. Introduction

There is an urgent welfare imperative to develop effective analgesic strategies for use on-farm to alleviate pain associated with livestock surgical husbandry procedures, including piglet castration [1,2,3,4,5]. Piglet castration is a common procedure conducted in commercial pig industries around the world with the aim of reducing boar taint [6], aggression and mating behaviours in male piglets [1]. Most commonly performed on-farm in the first week of life in piglets intended to be kept past sexual maturity, surgical castration, traditionally performed without anaesthesia or analgesia [1,4] induces evidence of pain and stress in piglets based on physiological responses, motor and vocal responses and alteration of piglet behaviour during and following the procedure [1,2,3,4,5,7,8,9,10,11,12,13,14,15,16,17]. 

Concern for the welfare of piglets undergoing castration is driving efforts to develop effective methods of pain mitigation. Although desperately needed, there are complexities developing safe and effective methods of pain alleviation applicable for on-farm use. Although reports vary [18,19,20,21], analgesics such as Non-Steroidal Anti-Inflammatory Drugs (NSAIDs) may be of use to address inflammatory-related pain that develops in the post-operative period [8,22], but generally lack efficacy to ameliorate pain during the procedure and in the early post-operative period when pain is most acute [4,23,24]. Standard methods for addressing surgical procedural pain, such as the use of general anaesthesia [18,25,26,27,28,29] or injected local anaesthetics, although effective to mitigate acute intra-operative pain, used alone, they provide little post-operative pain mitigation [22,24,25,28,30,31,32,33,34,35,36,37,38,39,40]. Furthermore, their use may be impractical and/or impeded on-farm by cost, logistics, occupational health and safety, food safety or welfare concerns. General or injected local anaesthetic interventions, for example, generally require skilled veterinary administration, may require specialised delivery and monitoring equipment, and/or extra labour which may be cost prohibitive [1,3,5]. General anaesthetics and sedatives may furthermore cause post-operative sedation, interfering with temperature regulation and feeding and increasing the risk of crush injury or mortality [1,41]. Additionally, anaesthetic and/or analgesic therapies may require time to take effect, requiring prolonged or double handling with associated increased stress [37]. Together, such factors may reduce the overall welfare benefit [1,2,3,4,5,6,7,8,41,42]. 

Topical local anaesthesia with Tri-Solfen^®^, (Animal Ethics Pty Ltd., Melbourne, Vic, Australia) a registered topical local anaesthetic and antiseptic formulation, applied intra-operatively by wound instillation, has recently emerged as an alternative farmer-applied method to deliver local anaesthesia and mitigate acute castration-related pain in lambs and calves, that is also safe and technically simple to administer in production conditions [35,36,43,44,45,46]. Tri-Solfen^®^ is a viscous liquid formulation containing a rapid onset local anaesthetic (Lignocaine hydrochloride 5%), a long-acting local anaesthetic (Bupivacaine hydrochloride 0.5%), along with a vasoconstrictor (Adrenalin acid tartrate (45 µg/L)) and an antiseptic (Cetrimide 0.5%). It was developed to provide single dose rapid onset local wound anaesthesia with a prolonged effect following application to surgical husbandry wounds in farm animals [43,44,45,46,47,48] and has regulatory approval for use to alleviate pain following castration and/or other husbandry procedures in lambs and calves in Australia and New Zealand. Recent studies suggest that it may be similarly effective to mitigate acute castration-related pain in piglets [44,48,49]. Lomax et al. [44], for example, reported intraoperative wound instillation of Tri-Solfen^®^ was effective to reduce wound hyperalgesic responses in piglets from within 1 min up to 4 h following castration. Amide local anaesthetics work by blocking pain signal conduction in nerve fibres. Applied topically to intact skin, they are of slow onset and limited efficacy due to inability to penetrate keratinised tissues to reach underlying nerve tissue. They may be rapidly effective (within 1 min) when applied to open wounds, where skin layers are disrupted and traumatised nerve fibres are exposed. They may be similarly rapidly effective, (within 30 s) [50,51], when applied to non-keratinised mucous membranes, which they can readily penetrate. In the setting of castration, Tri-Solfen^®^ is applied via wound instillation immediately following skin incision, thus coating the non-keratinised mucosal tissues of the spermatic cord, and the cut skin edge. Providing a 30 s dwell time may thus allow time for the anaesthetic actives to penetrate mucosal tissues of the spermatic cord, prior to applying traction on the testis and severing the spermatic cords. These latter steps are considered the most painful part of the procedure [14,15,52]. Consistent with this, reduced nociceptive motor and vocal responses during castration, (as well as reduce wound hyperalgesic responses in the first 2 h following castration) were recently reported in piglets receiving intraoperative wound instillation of Tri-Solfen^®^ with a subsequent 30 s dwell time [49]. To meet regulatory requirements and achieve approval for use, veterinary products must meet proof of efficacy requirements to internationally harmonised (VICH), Good Clinical practice (GL9) guidelines [53]. The current, follow-up regulatory drug field efficacy trial was undertaken to address these requirements. 

Proof of anaesthetic efficacy is challenging in neonatal piglets, particularly in the “field” or “on-farm” setting as per the VICH GL9 standard [53] required for veterinary drug regulatory approvals. There is no one “gold standard” or validated measure of “pain” in neonatal piglets. Instead, a range of predominantly indirect measures have been used in previous studies including physiological, motor and vocal responses, mechanical sensory testing and post-operative behavioural disturbances [54]. A pain scale has recently been validated for documenting post-operative pain in older piglets [55], however, at the time of these studies, was not available for neonatal piglets. Following a detailed review of the literature [54], piglet motor and vocal responses during castration, as well as direct wound sensory testing using von-Frey and needlestick stimulation, along with post-operative pain-related behaviour following castration, were identified as methods that had most consistently achieved these objectives. A range of other measures (including cortisol levels and physiological parameters) were excluded as lacking sensitivity or specific for pain, being inconsistent and/or unreliable measures of pain in castrated neonatal piglets, particularly in the setting of examining pain mitigation using local anaesthesia with adrenaline [54]. With regard to assessment of post-operative pain, the two selected variables are unable to be measured in the same cohort as wound sensory testing requires frequent post-operative handling, which interferes with the quiet observation required for behavioural assessment [44]. As the response to wound sensory testing post-castration was previously reported [44,49], the current study was designed to follow-up to investigate pain-related behaviour as the post-operative efficacy parameter. 

The detection of post-operative pain-related behaviour in neonatal animals is particularly challenging. A recent review of the literature [54] alongside others [56], revealed that behavioural changes post-castration in neonatal piglets may be subtle, transient and/or variably expressed, such that findings are not always reproducible, and, in some cases, contradictory results have been reported. Behavioural assessments usually involve either direct quiet observation and scoring of piglet behaviours by trained blinded observers, or continuous time-lapse video recording with off-line scoring either using event monitoring software or blinded observers. Assessments typically include observations of piglet (i) posture (e.g., lying, standing, sitting etc), (ii) location (e.g., under heat lamp, in contact with the sow or pen mates versus in isolation), and (iii) activities, including non-specific behaviours (e.g., sucking, sleeping, walking, playing, exploratory or aggressive behaviour, etc.) and “pain-specific” behaviours. This latter category, first detailed by Hay et al. [12] and based on pain-specific behaviours reported in other species, includes “prostration” (e.g., standing or sitting with head down below shoulder height), “huddled up” (i.e., ventral lying with three or more legs tucked up), “tremors or trembling”, “spasms (i.e., localised muscle spasm)”, “tail wagging” and “scratching” (i.e., rubbing the rump along the floor or walls, also called “scooting”). In view of the relatively low incidence and variability of pain-related behaviour, aggregation of “pain-specific behaviours” is commonly employed to derive a “total” or “global” pain score for each piglet over specific time periods [54]. Based on this recently published review [54], the most consistently reported variations in piglet behaviour following castration are an increase in total “pain-specific” behaviours. These are typically only evident during the earliest time period measured following castration (e.g., up to 180 min [54]), although much shorter durations (e.g., 30 min) have been reported [35]. The most consistently reported variation in behaviour in later time periods (e.g., 8–24 h+) is an increase in tail wagging and scratching.

The current study was undertaken to address drug regulatory proof of efficacy requirements to internationally harmonised (VICH), Good Clinical practice (GL9) guidelines [53]. Based on the data above, it was hypothesised that Tri-Solfen^®^ application by wound instillation followed by a 30 s dwell time would result in a significant reduction in acute pain-induced responses during subsequent castration and in the early post-operative period as compared with untreated castrated (control) piglets. Nociceptive motor and vocal responses to castration were selected as the primary and secondary efficacy variables for assessment of pain mitigation during castration, while the primary efficacy variable for evaluation of post-operative pain-mitigation was a significant reduction in total “pain-specific” behaviour in the first 30 min following castration, as assessed via a combination of focal assessments and scan sampling. 

## 2. Materials and Methods

### 2.1. Study Design

The study was a multi-centred, negatively controlled, randomised and blinded study conducted between June and August 2018 to test the clinical efficacy of Tri-Solfen^®^ (50 g/L Lignocaine hydrochloride, 5 g/L Bupivacaine hydrochloride, 0.048 g/L Adrenaline (as acid tartrate), 5 g/L Cetrimide). All study procedures were conducted under country-specific trial approvals, including ethical approval. In Germany, approval was obtained from the Schleswig-Holstein authority while approval for the Italian study was provided by the Ministero della Salute. The study was conducted by independent contract research providers, in accordance with VICH guidelines for conduct of regulatory safety and efficacy field trials with the principles of Good Clinical Practice (GCP) as laid down in the Council Directive 2001/82/EC and guideline CVMP/VICH/595/98 VICH Topic GL9 Step 7-Guideline on Good Clinical Practices (CVMP approved July 2000).

The animal study was performed, with owner consent, at two commercial pig farms in Germany and Italy dedicated to the management of breeding sows. Together, 173 commercially bred male piglets (Danish and Landrace × Large White) between 3 and 7 days of age and in good health were confirmed suitable for enrolment in the study. Prior power analysis indicated the requirement for a minimum of 80 animals per group to show a significant reduction at the 5% level. Litters with 6–12 male piglets (7 average) were selected for the study at each site. Piglets within a litter were individually identified by ear tag (applied on study Day −2) and spray/pen mark. Piglets within a litter were randomly allocated to treatment or control groups. All study piglets had standard blue spray-on piglet marker dye applied to the scrotum prior to procedures, to blind post-operative observers to any translucent-blue colouring that may have remained following application of treatment. This method of blinding was used rather than a placebo solution, to allow comparison with standard castration practice, and prevent confounding due to potential impacts (pain/inflammation/infection) from wound instillation of a placebo solution. Hence, the person performing the treatment was unblinded, however, this person did not participate in any trial assessments or data analysis.

Study animals were maintained in their normal farrowing pens as per standard farm practice with their dam and litter mates. Piglets had ad lib access to potable water and constant suckling access to their dam. Routine management practices were followed, however, procedures (such as tail docking) or other treatments which may have influenced the effect on potential pain responses or analgesia provided by treatment were not permitted and would have resulted in the removal of piglets from the study. 

Clinical examination was performed on Study Day −1 as part of piglet inclusion, prior to pre-operative behavioural assessments, as well as Study Day 1, after the morning behavioural assessments, and Study Days 6 and 12, in the morning. Examinations included measurements of weight and rectal temperatures using calibrated instruments and clinical assessment of general demeanour and vitality, in terms of normal/abnormal, and any evidence of other illness, injury, wound infection or inflammation. Additionally wound healing was scored on a scale from 1 to 6, based on Sutherland et al. [48].

Castration and treatment procedures to test the efficacy of this formulation were conducted as follows. Briefly, after the piglets were removed from their dams, each animal was restrained gently but firmly in a Kerbl piglet castration cradle (Albert Kerbl GmbH, Buchbach, Germany) to expose the ano-genital region of the piglet. The scrotal skin, (including underlying parietal tunica), was then incised to reveal and exteriorise the testis. In treated animals, Tri-Solfen^®^ was then applied with a total dose of 1 mL (piglets up to 2 kg), and 2 mL (piglets 2–4 kg) delivered via wound instillation using a 1 mL ball tipped applicator (Prodigy Instruments Pty Ltd., Sydney, Australia). Half of the total dose was applied to each side with care to ensure that the formulation coated the exposed spermatic cord and cut edges of the scrotal sack. A period of 30 s was allowed to elapse prior to the testes then being removed by severing the cord as per routine procedure. Piglets assigned to the control group were handled in the same way as the treatment group, but without the physical administration of Tri-Solfen^®^.

### 2.2. Video Recording during Castration and Nociceptive Motor Response Scoring

Video recording was performed using a device in fixed position above the cradle, to record post-treatment castration procedure and responses. A video-camera recording device (iPod Touch-Apple Inc. Cupertino, CA, USA) was used with the application ‘Timestamp Camera’. Each piglet was clearly identified by placing a number next to the piglet cradle within camera view. Video recording commenced following skin incision and product administration, approximately 10–20 s into the 30 s wait period prior to removal of the testes in animals from each group, documenting the spermatic cord severance and testicle removal phase and only finishing following the removal of the second testicle. 

The nociceptive motor response was assessed off-line by a blinded assessor, as recently described [8]. Briefly, piglet responses at four different time points were recorded: (1) traction on first testis; (2) cutting first spermatic cord; (3) traction on second testis; (4) cutting the second spermatic cord. A numerical rating scale (0–2) was used to grade piglet nociceptive motor response across all timepoints to give a total score of up to 8 for each piglet using the following grading increments: 0 = no motor response, 1 = mild motor response, such as short lived leg extension or front leg paddling or kicking but no major body resistance movement in the cradle, 2 = marked motor response, such as prolonged leg movements and/or marked body resistance movement in the cradle.

### 2.3. Audio Recording during Castration and Vocal Response Assessment

**Figure 1:**
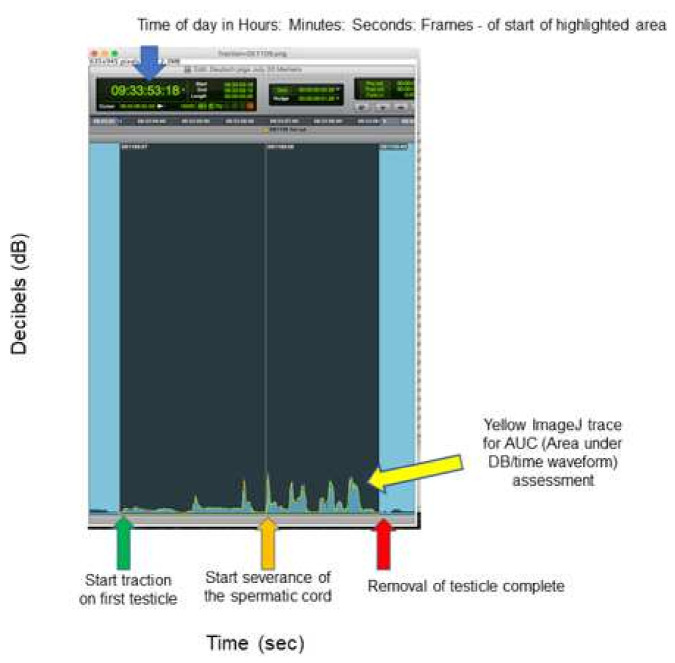
Representative screenshot of dB/time waveform, and yellow ImageJ tracing for AUC (area under the dB/time waveform) analysis. The image shows the black highlighted section encompassing the time from first commencing traction, until completing removal of the first testicle.

Sound was recorded with a validated Digital Sound Level Meter, positioned 60 cm directly in front of the snout. Recording commenced approximately 10–20 s into the 30 s wait period prior to removal of the testes in animals from each group through to 1–10 s after the cutting of the second cord. Sound files were analysed off-line by a sound consultant blinded to piglet treatment. Sound files were imported to Pro Tools^®^ (Avid Technology Inc., Burlington, MA, USA) and placed on the Pro Tools timeline at their time stamp position to enable synchronisation with the audio files from the video recording. Isolation and comparative quantification of piglet vocal output was achieved following annotation of the timeline with times for commencement of traction, and completion of cord severance for each testicle (Figure 1). Quantification was focussed on two time periods: (1) commencing traction to completion of severance of the first spermatic cord; (2) commencing traction to 1 s post-completion of severance of the second spermatic cord. Maximum vocalisation (decibels (dB), volume) for each time period was recorded. Screen shots were also generated with the same time duration window (X axis) and signal scale (Y axis). The Area Under the decibel (dB)/time waveform Curve (AUC) was then calculated (in pixels) using image analysis software (Image-J^®^ U. S. National Institutes of Health, Bethesda, MD, USA). This provides a measure of total vocalisation during each time period (Figure 1).

### 2.4. Assessment of Post-Operative Pain-Related Behaviour

The presence of pain-related behaviour in the piglets following castration was recorded by focal assessment and scan sampling using different independent trained observers blinded to treatment groups. Two separate observers were used, one for focal assessment and one for scan assessments, at each trial site. To minimise the potential for inter-observer error, the same observer performed all scan or focal assessments. 

Focal assessment was conducted for each piglet twice on Study Day −1 (morning and afternoon), and post-castration/treatment (Study Day 0 and Study Day 1) at approximately 1 min (+ 1 min), 15 min ± 2 min), 30 min (± 5 min) and then at approximately 60 and 90 min, as well as at 2, 3, 6, 8, 24 and 30 h. The 1 min focal assessment commenced following the return of the individual piglet to the pen post-castration, and included recording of the presence or absence of “pain-specific” behaviours using an ethogram based on Hay et al. [12], Moya et al. [13] and Gottardo et al. [35], including huddled up, stiffness, prostrated, attempts to suckle, scratching, tail wagging, tremors/trembling, panting (Table 1). Focal assessments were performed for a duration of 1 min with all activities recorded. Piglets were scored 1 = behaviour present; 0 = behaviour absent. Only individuals that demonstrated none of these at an assessment were recorded as not showing pain-associated behaviour at that assessment.

Scan sampling of animals was conducted over a three-hour period in the mornings and a two-hour period in the afternoons on the day prior to castration (Study Day −1), the day of castrations (Study Day 0) and the morning of the day after (Study Day +1). Morning scans on the day of castration/treatment were performed as soon as possible following the castration procedures being completed. During the scan sampling, behaviours were recorded at 10 min intervals with behavioural assessment conducted using an ethogram based on previous reports [12,13,35], including the general position, posture, activity and contact of each piglet, and including postures and activities deemed to be “pain-related” as per the definitions outlined in Table 2 (e.g., prostrated, stiff or hunched posture (standing or sitting), ventral lying in isolation, and walking with stiff or abnormal gait). For the scan assessments within each period, if an animal was recorded as demonstrating any of the following behaviours at any point during that period, then the animal was recorded as demonstrating pain-associated behaviour during that period. 

### 2.5. Statistical Methods

Raw data were entered into Microsoft Excel using double-entry techniques, and transferred into R software, with data labelled ‘Treatment Group 1’ and ‘Treatment Group 2’ maintaining blinding. Differences between treatment groups and study sites: any differences in weight between treatment group and study site, along with any evidence of an interaction between treatment group and study site were tested at the 5% level using a generalised linear model (GLM) with normally distributed outcome and linear link.

#### 2.5.1. Nociceptive Motor Response to Piglet Castration

The primary efficacy parameter for pain control during castration was a significant mitigation of the nociceptive motor response to castration. The scoring provided for the piglet’s motor response represented an ordinal score. At each of the four points, traction on the testis and cutting the spermatic cord for both the first and second testis, the motor response was graded on a scale of 0 to 2. Thus, the total score is within the range of 0–8. In view of the fact that a motor response in a piglet may occur in response to restraint rather than pain, it was considered that there was not a clear cut-off value indicative of ‘pain’. Instead, it was assumed that higher values either represent more pain or can be considered more likely when a piglet experiences a painful procedure. An ordinal regression was thus considered appropriate for these data, specifically an ordered probit model. The nociceptive motor response was analysed using an ordinal mixed effects regression model, on the ordinal behavioural response scale to estimate the effect of treatment, site and the interaction between treatment and site. Efficacy was determined by demonstrating statistically significant differences at the 5% level. Litter was fitted in this model as a random effect. 

#### 2.5.2. Vocal Response to Piglet Castration 

Vocal response was examined as a secondary efficacy parameter. The peak auditory response (peak dB) and total vocalisation (AUC) during spermatic cord cutting were analysed using a mixed effects linear model to estimate the effect of treatment, site, and the interaction between treatment and site. The natural logarithm of maximum volume (dB) and total volume of vocalisation (AUC) were taken to ensure normality in the model residuals. Peak vocalisation data were all negative, so these were multiplied by −1 before taking the natural logarithm.

#### 2.5.3. Post-Operative Pain-Related Behaviour 

The primary efficacy parameter for pain control following castration was based on the demonstration of a significant reduction in total specific “pain-related” behaviour by piglets observed during the first 30 min post-castration following return to pen. This involved assessment of all specific “pain-related” behaviours recorded via focal assessments at times, 1, 15 and 30 min, and/or via scan sampling assessments at times 0–10, 10–20 and 20–30 min (i.e., data from all focal and scan recordings in the first 30 min). For the focal assessments at each of these assessments, an animal was classed as showing pain-associated behaviour if it was recorded as having a huddled-up posture, stiffness, prostration, scratching the perineal area or trembling/tremors. Only individuals that demonstrated none of these at an assessment were recorded as not showing pain-associated behaviour at that assessment. For the scan assessments within each period, if an animal was recorded as demonstrating any one of the following behaviours at any point during that period then the animal was recorded as demonstrating pain-associated behaviour during that period: “Curved Spine (Hunched)”, “Head Down (Prostrate)”, “Leg Stiffness”, “Scratching scrotal/perineal area on floor”, or was recorded as “Sternal/Ventral lying” and isolated with activity “AI” (awake inactive). Using this approach, the large amount of information from the focal and scan observations was condensed for each individual into six binary observations (three focal results and three scan results) of pain-associated behaviour (yes/no). A generalised linear mixed model with binomially distributed outcome and logistic link was fitted to the presence or absence of pain-related behaviour data to estimate the effect of treatment, site, the interaction between treatment and site, time after castration and, where appropriate, the interaction of time and treatment. For all models, effects statistically significant at the 5% level were kept in the final models while others were removed by backwards elimination. Efficacy was determined by demonstrating either a statistically significant difference between treatments, across all time points at the 5% level, or a difference in reduction in pain-related behaviour over time, statistically significant at the 5% level.

#### 2.5.4. Additional (Secondary) Behavioural Assessments

Scan sampling data was analysed using multivariate analysis of variance (MANOVA) to estimate the effect of treatment, site, the interaction between treatment and site, time before/after castration and the interaction of time and treatment. Due to the large number of observations, the time of observations was considered in three phases: pre-castration, 0–30 min following castration, more than 30 min following castration. All observations made on each animal were retained in the data analysed. Effects significant at the 5% level were kept in the final model whilst others were removed by backwards elimination. For this analysis, behaviour was not categorised as “pain-associated” or not, but the multivariate nature of the observations was analysed (i.e., an animal could demonstrate several different behaviours within one time segment), hence the use of a MANOVA analysis. 

Post-hoc analyses of piglet pain-related behavioural responses pre- and post-castration, were conducted on scan sampling and focal assessment data separately to allow time and time x group comparisons. All time points before castration were set to a single time point (“−1” for the scan analysis, “Study Day −1” for the focal assessments), allowing improved precision of estimates of “normal” behaviour. This data was then fitted to a model with Time point and Treatment group as main effects and an interaction term between the two. This allowed the interpretation of the main effect of Treatment group as the difference between the two treatments to prior to castration, which was not expected to be different. The Time point effect is the difference between pre-castration and that time point for Group 1 (Tri-Solfen^®^) and the interaction term, the difference between the two groups at that time point, assuming no difference at the pre-castration time point. Initially a mixed effects model with Piglet ID as a random effect was fitted, but this failed to converge. Therefore, a GLM ignoring the effect of piglet was fitted. This was considered to be a conservative approach since one source of variability in the data, the differences between individuals, was not accounted for, thus increasing the residual variance in the model. This had the effect of increasing the minimum effect size that was considered to be statistically significant at the 5% level without altering the estimates of effect size. 

## 3. Results

### 3.1. Demographics and Clinical Parameters

From the 173 piglets, 86 piglets were assigned to the treatment group and 87 piglets were assigned to the untreated control group. Seven animals were subsequently removed from the trial, including (a) four animals from the treatment group (2 × intestinal prolapse; 1 × inadvertent variation in castration technique; 1 × treatment overdose); (b) three animals from the untreated control group (3 × inadvertent variation in castration technique). One animal in the control group died on Study Day 2. Necropsy performed by an independent pathologist revealed the piglet had a high grade diffuse, purulent fibrinous enterocolitis/peritonitis with *Escherichia coli* detected. A small number of individuals were removed from analyses of specific parameters due to incomplete data records (e.g., video recordings during castration failed in two control piglets).

There was a significant effect of study site on initial weight at the 5% level (*p*-value < 0.001), with mean body weight at the German site (2.72 kg ± 0.047) generally higher than those at the Italian site (1.97 kg ± 0.064), however, there were no difference in body weights between treatment groups at either site. 

There were no significant differences between treatment groups in demeanour and vitality parameters, rectal temperature, weight, or clinical wound assessments. There was a statistical difference in wound healing score with fewer animals in the treatment versus control group having a score < 5 at Day 6 (64/84 versus 80/85, respectively, *p* = 0.001) and <3 at day 12 (52/84 and 74/85, respectively, *p* < 0.001). There was one Serious Adverse Event in each group, including one death of a control piglet (due to peritonitis), and shock with recovery (due to anaphylaxis) in a piglet in the treatment group. There were no significant differences in adverse events between groups.

### 3.2. Piglet Nociceptive Motor Responses during Castration

Mean nociceptive motor response scores, were found to be higher in control piglets compared to Tri-Solfen^®^-treated piglets at each step of castration (e.g., traction, cutting), as well as in total scores (4.37 versus 2.86, Table 3). For the total nociceptive motor response scores, treatment group was found to be statistically significant at the 5% level, with the pain response predicted to be elevated in the untreated control group (*p* < 0.001). The results furthermore indicate the odds ratio of a nociceptive motor response score from an animal taken at random from the control group being in an equal or greater category to a random animal taken from the treated group was 2.9 × 10^−1.07^. 

### 3.3. Piglet Vocal Responses during Castration

**Figure 2:**
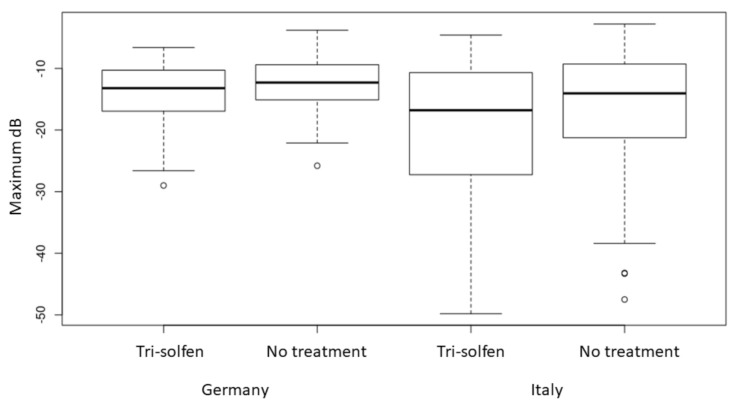
Box plot of peak vocal responses (peak dB) by treatment group and site. (Note, peak dB is recorded on a negative logarithmic scale where 0 is the maximum response). White circles are outliers.

**Figure 3:**
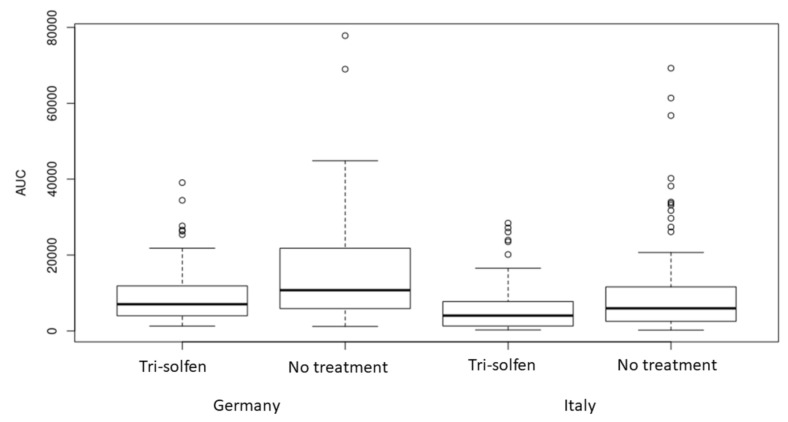
Box plot of total vocal responses (AUC) calculated from area under the dB/time waveform using image analysis (Pxls) (encompassing vocal response from commencing traction on the first spermatic cord until 1 s following removal of the second testicle in each piglet), displayed by treatment group and site. White circles are outliers.

The peak dB and total (AUC) vocalisation results for each treatment group can be visualised in Figure 2 and Figure 3 with the data for each experimental site presented separately. At both sites, greater peak intensity (dB) vocalisation results were recorded in the untreated control group compared to piglets treated with Tri-Solfen^®^, and this result was statistically significant at the 5% level (*p* < 0.0001) and consistent across sites (Figure 2). While differences in the peak intensity were noted between sites, these differences were not statistically significant at the 5% level (*p* = 0.064). Analysis of the AUC by treatment group revealed that vocal responses in the treatment group were statistically significantly lower than those of untreated piglets (*p* < 0.0001; Figure 3) during the traction and severance of each spermatic cord. This reduction was consistent across sites.

### 3.4. Pain-Related Behaviour in Piglets Following Castration

#### 3.4.1. Total “Pain-Related” Behaviour during the First 30 min Post-Castration

The GLM Model fitted to the pain-related behaviour during the first 30 min following castration found a difference in the odds of showing pain that was statistically significant at the 5% level (*p* < 0.0001) with the treated group, showing significantly less total specific “pain-related” behaviour than the untreated control group. Table 4 shows a greater number of Tri-Solfen^®^-treated piglets showed no pain behaviour (*n* = 23) in the 30 min post-castration versus their control group counterparts (*n* = 4). A piglet in the control group had the odds of showing “pain-related” behaviour which was 2.39 × 10^−0.87^ times that of a piglet in the treated group, across all observations. There was no evidence for an interaction between treatment and observation. In all observations, an animal in the untreated control group had higher odds of being observed exhibiting pain-associated behaviour than an animal in the treated group. 

Overall, the level of pain-associated behaviour decreased over the 30 min. Pain-related behaviour was more likely to be observed during the focal observations than the scan sampling, and this effect was statistically significant at the 5% level (*p* < 0.0001). A summary of the number of piglets exhibiting at least one pain-related behaviour during the focal and/or scan assessments performed over the 24 h prior to castration is also included in Table 4. A proportion of piglets exhibited at least one pain-related behaviour during general observations prior to castration, with the most common being “huddling up” and “tremors/trembling”. Note that these are not directly comparable with results from 0–30 min as pre-operative results include data from a greater scan assessment period (5 h). Proportions of piglets demonstrating stiffness, prostration or scratching during the Day −1 scans and focal assessments were very low (<5%). There were no statistically significant differences between groups.

#### 3.4.2. Scan Sampling Data

The secondary post-operative endpoint examined involved a comparison of the behaviour in treated and control groups by MANOVA, performed on the scan sampling data. Although statistical differences were found between scan results at the two sites and between the time periods (pre-treatment, first 30 min after castration and >30 min after castration; *p* < 0.01 for each), there was no treatment and time interaction (*p* = 0.475) or overall treatment effect (*p* = 0.39). Therefore, there was no effect shown on this secondary variable. 

**Figure 4:**
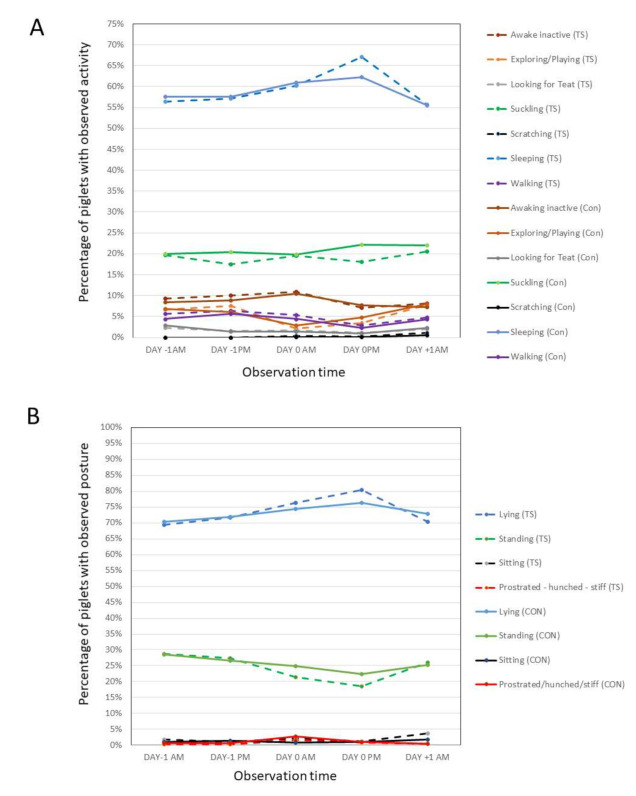
Results of scan assessments showing proportion of piglets (**A**) observed in different activities (**B**) observed in different postures in untreated control (CON) and Tri-Solfen (TS) treatment groups.

The scan sampling results (Figure 4) identified that, in the 24 h prior to castration, piglets were either sleeping or suckling in the majority of scan observations (55–60%, and ~20%, respectively). Smaller proportions were awake inactive, walking or exploring (5–10% of observations), with only minor proportions sitting, scratching or looking for teat (<5% of observations). There was an increase in instances of piglets sleeping in the first 24 h following castration as compared with pre-operative values. This was particularly evident over the two-hour Day 0 PM scan period (i.e., in the afternoon following castration). Consistent with an increase in the number of piglets sleeping, there was a relative decrease in the proportion of piglets awake inactive, walking and exploring, as well as a relative increase in piglets lying, and decrease in piglets standing during this same period, however, the proportion of piglets suckling was similar over all observation periods. The proportion of piglets displaying abnormal (pain-related) postures on scan sampling (i.e., showing prostration, standing or sitting, hunched standing, stiffness or scratching) was very low (<5% of observations) other than in the first 20 min following castration, during which time it was more prominent in untreated control than treated piglets. Statistical analysis of pain-related behaviour focused on data collected on Study Day −1 (pre-castration) and in 10 min intervals up to 2 h post-castration and identified no statistically significant difference in pain-related behaviour between the two groups prior to castration (data not shown). There was a low magnitude statistically significant increase (*p* < 0.05) in pain-related behaviours over time as compared with pre-operative values until the 40 min time point. This was most prominent 10–20 min post-castration in control animals, however, differences between groups did not reach statistical significance at any time point. The proportion of animals observed to be in the nest (under heat) or isolated varied between the two investigation sites. At all timepoints, a greater proportion of animals were observed to be in the nest (under heat) and a lesser proportion isolated (as opposed to in contact with the sow or littermates), at the German rather than the Italian site. 

#### 3.4.3. Focal Assessment Data

Post-hoc analysis was performed on the focal assessment data separately examining differences between groups and over time, including pre- and post-castration. No statistically significant differences were observed in total pain-related behaviours between treatment and control groups prior to castration. At the 1 min observation, the Tri-Solfen^®^-treated group showed a statistically significant increase in total pain-related behaviour (*p* < 0.05) as compared with pre-operative values. At no other time point was the level of pain-related behaviour significantly different from pre-castration levels in the treated group. The control group showed more total pain-related behaviour post-operatively compared with pre-operatively and as compared with the treated group at both 1 and 15 min timepoints post-castration. These differences were statistically significant at the 5% level.

## 4. Discussion

There is an urgent need for effective products for pain mitigation during and following piglet castration, that are practical for use in commercial production systems. To meet regulatory requirements and achieve approval for use, such products must meet proof of efficacy requirements to internationally harmonised (VICH) good clinical practice (GL9) guidelines [53]. In this study we report results of a VICH GL9 field efficacy study demonstrating efficacy of a topical local anaesthetic and antiseptic product (Tri-Solfen^®^), administered via intraoperative wound instillation, with a 30 s dwell time, to mitigate subsequent castration-related pain in piglets based on a significant reduction of nociceptive motor and vocal responses during the procedure and in post-operative pain-related behaviour evident in the first 30 min following the procedure. This builds on results from a previous study reporting efficacy based on a significant reduction in piglet motor and vocal responses during the procedure and wound-hyperalgesic responses in the post-operative period [49].

Reduced nociceptive motor response to the castration procedure (traction on and severing the spermatic cord) was the primary efficacy parameter for pain control during the procedure in this study. Studies of piglet nociceptive motor and vocal response identify traction on, and severing the spermatic cords [7,14,15] as inducing the greatest pain [7,14,15,16,52] of the procedure. Previous studies identified pre-emptive lidocaine or bupivacaine injection as effective to mitigate the pain-induced (nociceptive) motor response [22,28,36,37]. Most recently, Saller et al. [28] reported that, used in combination with light general anaesthesia to minimise the motor response to restraint and handling stress, Lidocaine 2%, Bupivacaine 0.5%, Mepivacaine 2% and Procaine administered via injection 20 min prior to castration were all effective to block the nociceptive motor response to castration as compared with those injected with normal saline (placebo). Limb movements were evident during injections, however, thereafter were virtually abolished in response to skin incision and to severing the cords in local anaesthetic treated, but not saline treated piglets, with the greatest effect in lidocaine and bupivacaine-treated piglets. Administered by injection such as into the testis, it takes 3 min or more for lidocaine to diffuse to the nerve tissue in the spermatic cord, and begin to take effect [57]. Onset may be much more rapid, within 30 s [50,51,58] however, when lidocaine is applied topically, directly to non-keratinised mucosal tissues such as in the conjunctiva or gingival sulcus. This appears also to apply to the spermatic chordal tissue in neonatal piglets as, using direct wound instillation following skin incision with a 30 s dwell time, it has been shown that Tri-Solfen^®^ was effective to mitigate the nociceptive motor response to the subsequent castration procedure, as compared with placebo-treated piglets, in a previous trial in 3–7 day old piglets undergoing castration on a commercial pig farm in Australia [49]. Consistent with this previous trial, in the current study, Tri-Solfen^®^ similarly administered, resulted in a significant reduction in mean nociceptive motor response to traction on, and excision of each testis during surgical castration. These results were consistent across both trial sites, and together with our previous study results [49], are considered to confirm the efficacy of the product used via this method to mitigate acute procedural castration pain in neonatal piglets.

Piglet vocal response to castration was examined as a secondary efficacy parameter for pain mitigation during castration in the current study, as in the previous trial [49]. Although piglets vocalise in response to restraint and handling, vocal responses in castrated piglets have previously been shown to be significantly louder and more frequent in control animals compared to those pre-treated with local anaesthetic [22,52] and sham-treated animals [14,59]. Analysis of the specific steps in the castration process revealed that the highest frequency calls are emitted when the spermatic cords are pulled and severed [15]. As noted in other publications investigating the role of local anaesthetic use to reduce pain in castrated piglets [22], a reduction in vocal responses during castration is considered to be indicative of significant pain mitigation. Sutherland et al. [48] reported no significant effect of topical anaesthetic (Tri-Solfen^®^ or Cetacaine^®^) instilled into the wound on piglet vocal response to castration, however, in this previous trial, no dwell time had been observed to allow the local anaesthetics to take effect. With inclusion of a 30 s dwell time, Sheil et al. [49] reported a significant reduction in vocal responses in Tri-Solfen^®^-treated piglets from the first traction on the first spermatic cord through to cord severance, compared to controls. This reduction in piglet vocal response was observed for both the maximum vocalisation result (peak dB) and the total vocal response, as measured by area under the decibel/time waveform. In this previous trial [49], a trend effect was also evident during traction on, and severing the second spermatic cord, however it did not reach statistical significance. Increased variability, associated with inadvertent prolongation of the analysed recording time, (which was continued until piglet vocalisation returned to baseline rather than ceasing after removal of the second testicle), was considered to have potentially confounded the results. In the current trial, the vocal response data analysis was confined to the specific time periods of traction on and severing each spermatic cord and ceased 1 s following completion of removal for each testicle. In this setting, and with greater power provided by a larger sample size, a significant reduction in piglet vocal response to severing both spermatic cords was evident and consistent with a reduction in nociceptive motor response during the same procedural events. The robustness of this observation is evidenced by the fact that this result was repeatable at two independent sites, working piggeries in Germany and Italy. These results are thus considered indicative of significant procedural pain mitigation in Tri-Solfen^®^-treated piglets.

It should be noted that the nociceptive motor or vocal response to skin incision was not measured as Tri-Solfen^®^ was only applied following incision and is not expected to have a pain mitigating effect. Ideally, in the future, methods of concurrently providing pre-incisional skin anaesthesia, may be developed that are practical, rapid and painless. One such option, is cryo-anaesthesia, which is currently under investigation as a method of pre-operative skin anaesthesia for husbandry procedures in piglets and calves [60,61]. Topical anaesthesia can also be effective to anesthetise intact skin prior to minor procedures, however, may take 20–30 min to have effect due to difficulty penetrating keratinised tissues, which may not be practical for on-farm use. Subcutaneous injection of a local anaesthetic provides another option for pre-incisional skin anaesthesia. It is notable, however, that the injections themselves are painful, and may be equally as painful as quick skin incision, based on similar nociceptive motor [28], and vocal response scores [62,63]. This may also increase the total stress of the procedure due to the need for either doubling handling or prolonged restraint for 3 min to reach skin anaesthetic effect, such that it is not clear that it would provide an overall welfare benefit in this setting [37]. In the interim, it is notable that skin incision is consistently identified as the least painful part of the castration procedure [14,15,36,63,64] and anaesthetic onset is very swift (within one minute) following Tri-Solfen^®^ application topically to the incision site, as the local anaesthetics actives are able to act directly on exposed traumatised nerve endings in the cut skin edge, as noted above. Using Von-Frey and needlestick assessment at the cut skin edge, rapidity of onset of anaesthetic effect (within one minute) has been demonstrated following Tri-Solfen^®^ application to a range of wounds, including the piglet castration wound [43,44], as well as husbandry wounds in lambs and calves [43,45,65].

In the current study, an acute pain-related behaviour response, evident in control piglets in the first 30 min post-castration, was significantly reduced in Tri-Solfen^®^-treated piglets. The results of the behavioural analysis appear to further confirm previous reports from direct observational studies [8,12,13,19,35,66] identifying that an acute increase in “pain-specific” behaviours occur in neonatal piglets in the early time periods following castration. These behaviours are subtle and short-lived, being evident at significant levels in this study primarily only over the first 30 min (scan and focal assessments data combined) and at 1 and 15 min post-castration using focal assessment data alone. Focal assessments proved to be more sensitive than scan sampling for detection of pain-associated behaviour. Scan sampling revealed the very low proportion of piglets exhibiting such behaviours. Similar results were also previously noted in piglets receiving pre-emptive injectable NSAIDs, or pre- and post-castration topical preparations of tetracaine [35], in which piglets castrated without treatment showed increased frequency of specific pain- related behaviour in the first 30 min in comparison with sham-handled and treated piglets, however, no behavioural differences were apparent after 60 min.

Scan data revealed the majority of piglets were observed to be sleeping or suckling during scan observations, and the proportion sleeping increased significantly the afternoon following castration. This may explain the difficulty in detecting pain-associated behaviour in piglets of this age over this time period. Increased piglet sleeping, as was evident at around 6–8 h following castration in the current trial, has also been reported by Viscardi and Turner [63,67,68] who similarly compared piglet behavioir pre- and post-castration, and also by Kluivers and Poodt [8], who reported 70–75% of piglets sleeping during scan assessments the afternoon following castration or sham handling, with no difference between groups. A sleep response to aversive stimulation is known to occur in neonates [69,70]. As handling and restraint are aversive to piglets, increased sleeping following handling and restraint may be common to both castrated and sham-handled animals and requires further investigation. In this setting, trends for increased lying, with reduced standing, walking, exploring, etc., could all be consequent upon an increase in piglets sleeping following handling, rather than being indicative of pain post-castration. Interestingly, however, the increase in sleeping behaviour did not appear to effect suckling or nursing behaviour pre- and post-castration.

From the focal assessment data, there were no significant differences in tail wagging or scratching pre-castration or in the early hours post-castration in piglets in this trial. There was an increase of relatively low magnitude in both groups at later time points with a peak at 24 h, however. This observation is consistent with Hay et al. [12] and Viscardi and Turner [63,67]. Tail wagging and scratching are generally considered to indicate irritation or discomfort and have been reported to peak 24 h following castration [12,63,67], and have been reported to remain elevated for several days in some studies [12,64]. Others, however, have not reported an increase in tail-wagging post-castration [13]. Paradoxically, tail wagging has been reported to be increased in piglets treated with injected lidocaine administered pre-castration [8] or topical lidocaine spray applied post-castration [19], as opposed to untreated castrated controls. Authors of these studies hypothesised that lidocaine may induce a sensation as it wears off (such as tingling) that induces tail wagging, or the 80% alcohol content of the spray-formulation use may have sensitised the tail. Tri-Solfen^®^ contains 5% lidocaine, along with 0.5% Bupivacaine. No significant increase in tail wagging in piglets in the first hours after castration as compared with pre-operative values was observed, nor a significant difference in tail wagging between treated and untreated control piglets in the current trial. Bupivacaine has a slower onset of action than lidocaine, generally 5–10 min when applied to mucosal tissues, however, it has a more prolonged duration of action, lasting up to several hours. It is possible that the action of bupivacaine prevents any sensation due to lidocaine wearing off, and thus prevents increased tail wagging in this situation.

It should be noted that acute pain-related behaviour was only evident in piglets for the first 30 min in this study. This is of significantly shorter duration than acute post-operative mechanical hyperalgesia, which has been documented from 2 to 4 h post-castration in untreated neonatal piglets using response to von-Frey and needlestick stimulation of the wound [44,49]. This is not unexpected as quantitative mechanical sensory testing examines evoked pain responses, which may or may not reflect the degree of “spontaneous” pain an animal experiences from a wound site in the absence of a stimulus (e.g., while lying quietly or sleeping). Kawamata et al. [71], for example, subjected human volunteers to a small incision in the volar forearm, an area frequently used for sensory testing in humans. In these volunteers, pain at rest decreased and disappeared within 2 h after the experimental incision. Pain response to mechanical stimuli at the incision (i.e., primary mechanical hyperalgesia), however, was present for several days. 

In the field, safety was evident in the lack of impacts on weight, demeanour, vitality, rectal temperature or incidence of adverse events including wound infection. Differences in wound healing scores suggest possible minor delay in wound scab resolution in treated piglets in this trial, however, this did not appear to have a significant impact on clinical recovery. Initial wound size was not measured in this study to know if there may have been an inadvertent difference at the time of surgery. Wound healing differences were not apparent in Tri-Solfen^®^-treated piglets in a previous trial reported by Sutherland et al. [48], or in a regulatory Target Animal Safety study (VICH GL43) [72], to be reported separately. Similarly, no significant impacts on wound healing and/or improved wound healing have been reported following use of Tri-Solfen^®^ on other wound types in other species [47,73,74,75].

## 5. Conclusions

The results of this VICH GL9 regulatory drug field efficacy study are considered to confirm that intra-operative administration of Tri-Solfen^®^ via wound instillation immediately following skin incision and then a 30 s wait period results in significant mitigation of piglet pain during the subsequent castration procedure, and the early post-operative period, when pain is most acute. As topical wound instillation of Tri-Solfen^®^ (with 30 s dwell time) may be farmer applied and involves relatively minimal interruption to the standard procedures utilised for piglet castration, it is anticipated that it may provide an important and viable strategy to mitigate acute castration-related pain in piglets under commercial production conditions, and contribute to improve piglet welfare where surgical castration is still utilised in commercial pig facilities worldwide.

## Figures and Tables

**Table 1:** Description of variables included in focal assessment of pain-related behaviours.

Behaviour	Description
Huddled up	Lying with at least three legs tucked under
Stiffness	Lying or standing with extended and tensed legs
Prostrated	Awake, sitting or standing motionless, with the head down, lower than shoulder
Attempts to suckle	Attempts to suckle by walking and pushing other piglets while most of the others are suckling
Scratching	Scratching the scrotal/perineal area by rubbing it against the floor or the pen wall
Tail wagging	Tail movements from side to side or up and down
Tremors/Trembling	Shivering as with cold. The animal may be lying, sitting or standing
Panting	Higher respiratory rate and superficial respiration

**Table 2:** Ethogram including description of variables included in scan sampling of general and pain-related behaviours in piglets post-castration in this study.

Behaviour	Description
Isolated or in contact with littermates	Isolated was defined as being aside from the other piglets alone or with one piglet at most. A distance of at least 40 cm (approximately the width of two piglets) separated the piglet from the closest littermate.
	In contact was defined as being near at least two piglets with a distance of less than 40 cm.
Position: crate or nest	Crate (piglet is out of the nest area)
	Nest (under the cone of light of the lamp with at least half of its body)
Posture: standing, lying or sitting	Standing - Head down (prostrate). Body weight supported by the four legs, piglet awake, standing motionless or waling with the head down, lower than shoulder level; OR - Curved spine (hunched). Body weight supported by the four legs, awake, standing motionless or walking with a curved spine (kyphosis); OR - Leg stiffness. Standing or walking with extended and tensed legs (standing or walking on tiptoes); OR - Normal. Body weight supported by the four legs. No head prostrated, no curved spine and no leg stiffness.
	Lying - Group/Flock. The piglets were close and disorderly overlapped with each other and the lying posture was not classifiable; OR - Sternal/Ventral. Lying sternal, bodyweight supported by belly, sternum in contact with the floor; OR - Lateral. Lying lateral, body weight supported by side, shoulder and ribs in contact with floor.
	Sitting - Sitting head down (prostrate). Body weight supported by hind quarters flexed with ischium on the floor and front legs extended. Awake, sitting motionless, with the head down, lower than shoulder level; OR - Normal. Body weight supported by hind quarters flexed with ischium on the floor and front legs extended, head not down/lower than shoulder level.
Activity (explorative behaviours)	Awake inactive. No specific activity but awake. Lying sitting or standing; OR
	Suckling. Teat in mouth, vigorous and rhythmic suckling movements; OR
	Looking for teat. Attempts to find a teat and to suck. The piglet is desynchronised from the suckling behaviour of the other piglets; OR
	Exploring/Playing. Piglet nosing/manipulating: the snout is close to or in contact with a substrate or littermate. Snout movements may be observed. Playing. Head shaking, springing (sudden jumping or leaping), running with vertical and horizontal bouncy movements. Can involve littermates (gentle nudging or pushing, mounting, chasing); OR
	Aggression. Forceful fighting, pushing with head or biting littermates in a violent manner; OR
	Walking. Slowly moving forward with one leg at a time; OR
	Sleeping. Lying down, eyes closed.

**Table 3:** Mean (± standard deviation) nociceptive motor response during piglet castration between Tri-Solfen^®^ treated and untreated control groups.

Treatment Group	Traction on First Testis ^a^	Cut First Spermatic Cord ^a^	Traction on 2nd Testis ^a^	Cut Second Spermatic Cord ^a^	Total Motor Response Score ^b^
Tri-Solfen^®^	0.1 (±0.4)	1.3 (±0.7)	0.4 (±0.6)	1.1 (±1.1)	2.9 (±1.5)
Control	0.6 (±0.7)	1.7 (±0.5)	0.5 (±0.7)	1.6 (±1.6)	4.4 (±1.5)

^a^ Maximum response score of 2; ^b^ Maximum response score of 8.

**Table 4:** Number of piglets showing specific pain-related behaviours pre-castration over a total 5 h observation period and 0–30 min post-castration.

	Pre-Castration ^a^		0–30 Min Post-Castration	
Total observation (time period)	5 h		0.5 h	
Group	**Tri-Solfen** **(*n* = 84)** ***n* (%)**	**Control** **(*n* = 86)** ***n* (%)**	**Tri-Solfen** **(*n* = 83)** ***n* (%)**	**Control** **(*n* = 86)** ***n* (%)**
Huddled	39 (46.2)	38 (44.2)	37 (44.6)	52 (60.5)
Stiffness	1 (1.2)	0 (0.0)	22 (26.5)	27 (31.4)
Prostrated	3 (3.6)	3 (3.5)	30 (36.1)	41 (47.7)
Scratching	0 (0.0)	1 (1.2)	2 (2.4)	0 (0.0)
Tremors	9 (10.7)	5 (5.8)	11 (13.3)	16 (18.6)
Piglets showing at least 1 pain-related behaviour: focal assessment	46 (54.8)	46 (53.5)	59 (71.1)	78 (90.7)
Piglets showing at least 1 pain-related behaviour: scan sampling	24 (28.6)	25 (29.1)	12 (14.5)	33 (38.4)
Piglets showing at least 1 pain-related behaviour: focal Assessment OR Scan Sampling	57(67.9)	55 (64.0)	60 (72.2)	82 (95.3)
Piglets not exhibiting any pain-related behaviour	27 (32.1)	31 (36.0)	23 (27.7)	4 (4.7)

^a^ Numbers shown are from the intention to treat population.

## Data Availability

Relevant data is contained within the article.
